# *Fsp1*-Mediated Lineage Tracing Fails to Detect the Majority of Disseminating Cells Undergoing EMT

**DOI:** 10.1016/j.celrep.2019.10.107

**Published:** 2019-11-26

**Authors:** Laura Bornes, Roan Hugo van Scheppingen, Evelyne Beerling, Tim Schelfhorst, Saskia Inge Johanna Ellenbroek, Danielle Seinstra, Jacco van Rheenen

**Affiliations:** 1 Division of Molecular Pathology, Oncode Institute, Netherlands Cancer Institute, Amsterdam, the Netherlands

**Keywords:** epithelial-to-mesenchymal transition, EMT, metastasis, cancer, plasticity, epithelia, mesenchymal, dissemination, E-cadherin, fsp1

## Abstract

Epithelial-to-mesenchymal transition (EMT) has long been thought to be crucial for metastasis. Recently a study challenged this idea by demonstrating that metastases were seeded by tumor cells that were not marked by an EMT lineage-tracing reporter on the basis of the expression of the mesenchymal marker *fsp1*. However, the results of this study and their interpretation are under debate. Here, we combine the lineage-tracing reporter with our real-time EMT-state reporter and show that the *fsp1*-based EMT lineage-tracing reporter does not mark all disseminating mesenchymal cells with metastatic potential. Our findings demonstrate that *fsp1*-mediated lineage tracing does not allow any conclusions about the requirement of EMT for metastasis. Instead our data are fully consistent with previous reports that EMT is not a binary phenomenon but rather a spectrum of cellular states.

## Highlights

• Combination of historical and dynamic EMT reporter
• Not all cells undergoing EMT are labeled by the *fsp1*-mediated lineage-tracing mark
• The pool of mesenchymal cells that are not traced by *fsp1* have metastatic potential
• EMT is a spectrum of cell states and gene expressions rather than a binary process

## Graphical Abstract

**Figure:**
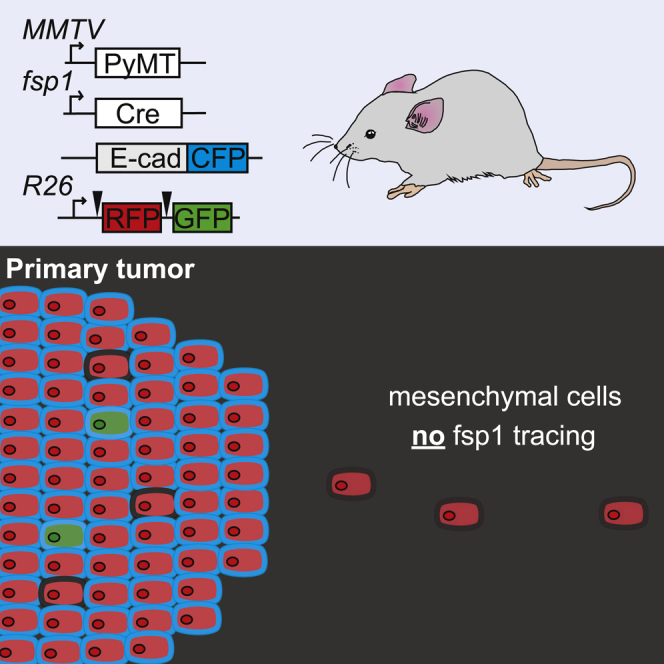

## Teaser

Bornes et al. use a combination of *fsp1*-based lineage-tracing and E-cadherin-based real-time EMT-state reporter. They demonstrate that the majority of disseminating mesenchymal cells in breast tumors are not labeled by the *fsp1*-mediated lineage-tracing mark but do have metastatic potential. These findings uphold the role of EMT in metastasis.

## Introduction

Cancer is difficult to treat when cells from the primary tumor spread to other sites of the body to form distant metastases. The metastatic cascade is a multi-step process including detachment from neighboring cells, movement to and entry into the circulation, exit from the circulation, and colonization of a secondary site (Hanahan and Weinberg, 2011). It has long been speculated that metastatic cells acquire disseminating and stem cell properties by hijacking a developmental program called epithelial-to-mesenchymal transition (EMT) (Cano et al., 2000, Mani et al., 2008, Nieto et al., 2016, Tsai et al., 2012, Yang et al., 2004). Cells that undergo EMT often decrease expression of epithelial proteins such as adherent junction molecule E-cadherin (E-cad) and frequently gain expression of mesenchymal proteins such as fibroblast-specific protein 1 (Fsp1) (Nieto et al., 2016, Thiery et al., 2009). The potential crucial role of EMT in acquiring invasive and metastatic properties, and even the very existence of EMT in unperturbed tumors, is heavily debated (Bill and Christofori, 2015, Brabletz et al., 2018, Diepenbruck and Christofori, 2016, Jolly et al., 2017, Ye et al., 2017, Yeung and Yang, 2017). We previously demonstrated that EMT exists in unperturbed tumors *in vivo*, by generating a mouse model for ductal mammary carcinomas (on the basis of the expression of polyoma middle-T antigen [PyMT]) in which endogenous E-cad is fused to monomeric CFP (mCFP). Using flow cytometry, we identified a small population of cancer cells in which E-cad is downregulated and all classical mesenchymal markers are upregulated, a population we refer to as E-cad^LO^ cells. Using flow cytometry, intravital microscopy, and transplantation assays, we identified that E-cad^LO^ cells can disseminate and upon arrival at a distant site revert to an epithelial state to seed metastases, thereby providing direct evidence for the existence of EMT in unperturbed tumors (Beerling et al., 2016). However, the commonly assumed crucial role of EMT in metastasis has recently been challenged in a study in the same PyMT-breast cancer model. In this study, an elegant EMT lineage-tracing reporter was developed that permanently marks cells fluorescently upon expression of the mesenchymal protein Fsp1. Strikingly, the authors found that the vast majority of metastases were negative for this genetic inheritable mark (Fischer et al., 2015). Therefore, it was concluded that these metastases are seeded by disseminating cells that are in an epithelial state rather than a mesenchymal state, which strongly challenges the idea that EMT is crucial for metastasis. However, this interpretation and conclusion hold true only if all cells that become mesenchymal are marked by *fsp1*-mediated lineage tracing. This has been challenged in many reports and reviews (Aiello et al., 2017, Aiello et al., 2018, Bill and Christofori, 2015, Brabletz et al., 2018, Diepenbruck and Christofori, 2016, Jolly et al., 2017, Reichert et al., 2018, Ye et al., 2017, Yeung and Yang, 2017). For example, Ye et al. (2017) showed using immunofluorescence staining of PyMT tumor sections that only a small fraction of mesenchymal cancer cells positive for Zeb1 or Snail also express Fsp1. To further investigate this, we here combine the *fsp1*-based lineage-tracing reporter with our real-time E-cad-based epithelial-mesenchymal state reporter and further characterize the disseminating cells of metastatic PyMT-mediated mammary tumors.

## Results and Discussion

**Figure 1:**
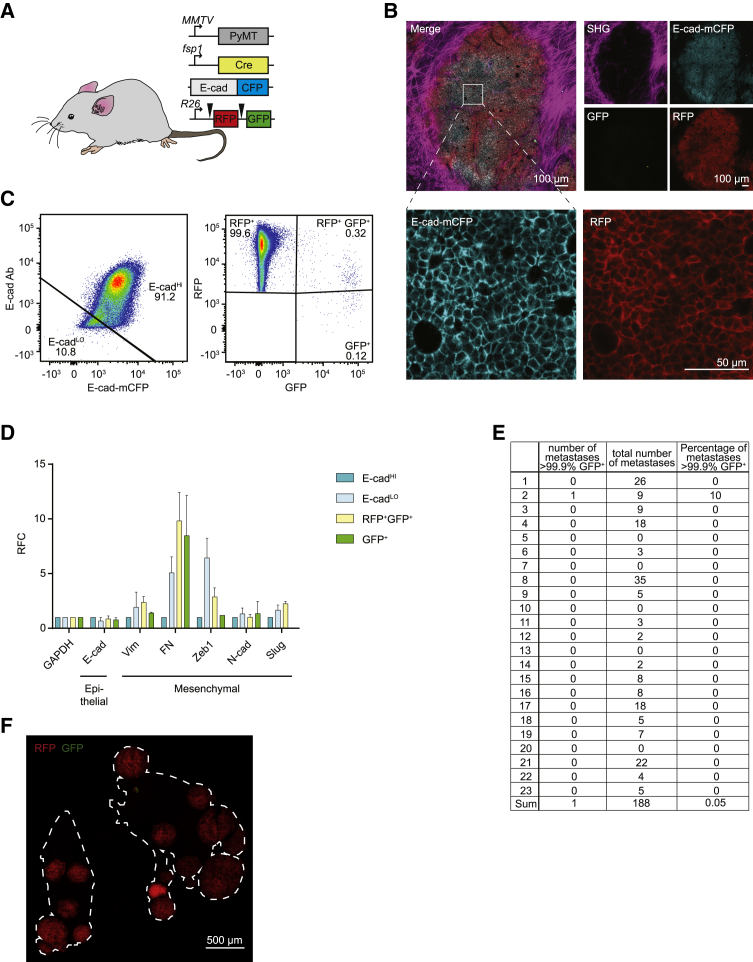
Epithelial-to-Mesenchymal Transition Reported by Historical Lineage-Tracing Reporter and Current E-Cadherin Status in Murine Metastatic Mammary Carcinoma Model (A) Schematic of fluorescent transgenic mouse model, carrying polyoma middle-T (PyMT) oncogene driven by the mammary gland specific *MMTV* promoter, Cre recombinase under the control of the *fsp1* promoter, endogenous E-cad labeled with mCFP, and ubiquitous expression from the *R26* locus of membranous RFP-STOP, which is flanked by *lox*P sites, leading to excision and subsequent expression of membranous GFP upon Cre expression. (B) Top panel: representative multi-photon images of the fluorescent PyMT mammary tumors from recipient mice. Scale bar, 100 μm. Bottom panel: high-magnification images of the E-cad-mCFP and RFP channel of the boxed area in the top left image. Scale bar, 50 μm. (C) Representative scatterplot from flow cytometry analysis of primary tumor of recipient mice for either absence of endogenous E-cad-mCFP expression and antibody staining (E-cad^LO^) or presence of high expression of E-cad and staining for Ab (E-cad^HI^ tumor cells) or subdivision of RFP^+^ or GFP^+^ cells. (D) Relative mRNA expression of classical EMT marker genes (E-cad, Vimentin [Vim], fibronectin [FN], Zeb1, N-cad [N-cadherin], Slug) determined using qPCR of sorted RFP^+^E-cad^Hi^, RFP^+^E-cad^LO^, RFP^+^GFP^+^, and GFP^+^ cells from primary tumors. Plotted mean and SD of n = 3 mice. (E) Quantification of lung metastases of all transplanted mice. (F) Representative multi-photon image of metastases in lung secetion from recipient mice. Outlining of lung tissue is shown by white dashed line. Scale bar 500 μm.

To further characterize the EMT status of disseminating cells, we crossed the EMT lineage-tracing mouse model used by Fischer et al. (2015) (*MMTV*-PyMT; *fsp1*-Cre; *R26*-mTmG) with our real-time E-cad-based EMT reporter (E-cad-mCFP) (Figure 1A). The resulting mice (*MMTV*-PyMT; *fsp1*-Cre; *R26*-mTmG; E-cad-mCFP) spontaneously develop mammary tumors that resemble invasive ductal carcinoma (Lin et al., 2003). All cancer cells in these tumors ubiquitously express membrane-targeted Tomato (from here on referred to RFP^+^), which is lost upon Cre-mediated recombination, concomitantly leading to gain of membrane-targeted GFP (GFP^+^) (Figure 1A). Expression of the Cre recombinase is driven by the promoter of the mesenchymal protein *fsp1* to genetically and inheritably mark cells that have been in a mesenchymal state (Fischer et al., 2015, Zheng et al., 2015). Last, in all epithelial cells (including cancer cells), the endogenous E-cad is tagged with a mCFP, which is delocalized from the membrane or lost upon EMT (Beerling et al., 2016; Figure 1A).

In order to determine whether previous reported results can be recapitulated in this new mouse model, we established primary organoid cultures from highly aggressive tumors and isolated the epithelial population of cancer cells (i.e., E-cad-mCFP^+^; RFP^+^; GFP^−^ cells). As we have previously demonstrated (Beerling et al., 2016), orthotopic transplantation of these epithelial cancer cells in recipient mice results in primary tumors that are morphologically indistinguishable from the original tumor and metastasize to the lungs spontaneously (Figure 1B; Table S1 for all details on the mice included in this study). Importantly, because the healthy cells of these recipient mice are not fluorescent, epithelial and mesenchymal cancer cells can be distinguished on the basis of the expression of E-cad-mCFP, GFP, and RFP. We observed that the majority of cancer cells in primary tumors were in an epithelial state (i.e., E-cad-mCFP^+^ cells, from here on referred to as E-cad^HI^ cells) and expressed classical epithelial markers. In addition to E-cad^HI^ cells, we found a much smaller population of E-cad^LO^ cells (on average <5%) (Figure 1C). This percentage was higher than we have reported before, most likely because of a change of our mouse facility, different flow cytometry filters, and potentially the aggressive nature of the donor tumors. Indeed, we observed a relationship between the number of E-cad^LO^ cells in the tumor, which is related to the amount of lung metastases (Figure S1). Importantly, and in line with our previous findings, E-cad^LO^ cells were truly mesenchymal cells, as they expressed all classical mesenchymal markers (Figure 1D). Last, in line with previous findings (Fischer et al., 2015, Zheng et al., 2015), we observed that the vast majority of lung metastases were in an epithelial state (i.e., E-cad-mCFP^+^) and were not marked by the *fsp1*-mediated EMT lineage-tracing reporter (i.e., RFP^+^ instead of GFP^+^) (Figures 1E and 1F), indicating that these cells never expressed *fsp1*.

**Figure 2:**
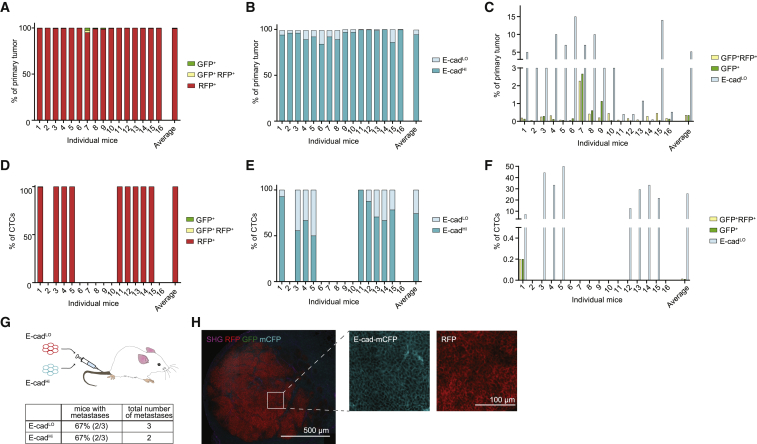
Mesenchymal E-cad^LO^ Population Exceeds *fsp1*-Lineage-Traced Population in Primary Tumor and Circulation and Has Metastatic Potential (A) Percentage of GFP^+^ (green), GFP^+^ RFP^+^ (yellow), and RFP^+^ (red) tumor cells in primary tumors of recipient mice. n = 16 mice. (B) Percentage of E-cad^HI^ (blue), E-cad^LO^ (light blue) tumor cells in primary tumors of recipient mice. n = 16 mice. (C) Comparison of percentage of E-cad^LO^ (light blue) with GFP^+^ (green) and GFP^+^ RFP^+^ (yellow) tumor cells in primary tumors of recipient mice. n = 16 mice. (D) Percentage of GFP^+^ (green), GFP^+^ RFP^+^ (yellow), and RFP^+^ (red) circulating tumor cells collected from the right cardiac chamber of recipient mice. n = 16 mice. (E) Percentage of E-cad^HI^ (blue), E-cad^LO^ (light blue) circulating tumor cells collected from the right cardiac chamber of recipient mice. n = 16 mice. (F) Comparison of percentage of E-cad^LO^ (light blue) with GFP^+^ (green) and GFP^+^ RFP^+^ (yellow) circulating tumor cells collected from the right cardiac chamber of recipient mice. n = 16 mice. (G) Top panel: schematic overview of experimental setup: fluorescence-activated cell sorting (FACS)-sorted E-cad^HI^ or E-cad^LO^ cells of orthotopically transplanted mice were injected in the tail vein of acceptor mice. Bottom panel: quantification of lung metastases of all transplanted mice. n = 3 mice per condition. (H) Representative multi-photon image of lung metastasis from (G). Scale bar, 500 μm. Right panels: zoom of boxed area showing detected E-cad-mCFP and RFP signal. Scale bar, 100 μm.

Although the lack of GFP^+^ metastases was previously interpreted as evidence for the lack of requirement of EMT for seeding metastases, this interpretation holds true only if all cells undergoing EMT are genetically and inheritably marked. To test this, we isolated tumor cells from primary tumors on the basis of the presence of GFP, RFP, and membranous E-cad using flow cytometry (Figure 1C). In line with microscopy analyses (Figure 1B) and previous data (Beerling et al., 2016), the majority of cancer cells were E-cad^HI^ and RFP^+^, and only a small number of cells were positive for GFP (Figure 1C). Similar to E-cad^LO^ cells, the GFP^+^RFP^+^ and GFP^+^ cells also expressed mesenchymal markers (Figure 1D). Although there was a large variation among individual mice, the percentage of GFP^+^ cells was on average 0.3%, while that of mesenchymal E-cad^LO^ cells was on average 5% (Figures 2A–2C). From these data, we conclude that the *fsp1*-based lineage-tracing reporter marks only a minor fraction of all mesenchymal cells in the primary tumor. To test whether this holds true not only for primary tumor cells but also for disseminating cells, we isolated circulating tumor cells (CTCs) from the right heart chamber and analyzed them using our flow cytometer strategy. Despite a large variation regarding the number of CTCs in each individual, 25% of all disseminating cells were in a mesenchymal state (E-cad^LO^), while only 0.01% were GFP^+^ (Figures 2D–2F). Importantly, when we injected mesenchymal E-cad^LO^ cells into the circulation, these cells formed E-cad^+^ metastases, illustrating that these cells were plastic and could seed epithelial metastases (Figures 2G and 2H). Combined, our data show that the vast majority of disseminating cells in a mesenchymal state that have metastatic potential are not marked by the *fsp1*-based EMT lineage-tracing reporter.

### Final Remarks

It has been extensively hypothesized that EMT is crucial for metastasis (Hanahan and Weinberg, 2011, Nieto et al., 2016, Thiery et al., 2009). This idea was challenged by observations that metastases can be seeded by cells that have not expressed *fsp1*-driven Cre recombinase at any point during the metastatic cascade (Fischer et al., 2015, Fischer et al., 2017, Zheng et al., 2015). This observation only disputes a crucial role of EMT for metastasis, if all cells undergoing EMT are labeled by the *fsp1*-mediated lineage-tracing mark. In line with previous studies (Aiello et al., 2017, Aiello et al., 2018, Bill and Christofori, 2015, Brabletz et al., 2018, Diepenbruck and Christofori, 2016, Jolly et al., 2017, Reichert et al., 2018, Tsai et al., 2012, Yang et al., 2004, Ye et al., 2017, Yeung and Yang, 2017, Yu et al., 2013), we show that this is not the case. Instead, we find that the vast majority of mesenchymal cells in primary tumors and during dissemination are not marked by this historical *fsp1* marker. Therefore, the lack of metastases that are seeded by cells that express *fsp1* does not necessarily mean that EMT is not required during the metastatic cascade. A potential explanation is the occurrence of partial EMT, resulting in the presence of hybrid cells that display some characteristics of both epithelial and mesenchymal states. Indeed, different transition states (i.e., partial EMT) have recently been identified (Aiello et al., 2018, Pastushenko et al., 2018). In line with this idea, recent studies in pancreatic cancer have shown that partial EMT is important for metastasis formation in specific organs (Reichert et al., 2018). Our data are fully consistent with previous findings that EMT represents a spectrum of different cellular states, rather than a binary phenomenon, each of which may have different roles during the metastatic cascade. So, despite a lack of direct evidence for a crucial role of EMT in metastasis, our data illustrate that the suggestion that EMT is required for metastasis is less controversial than recently assumed and stated.

## STAR★Methods

### Key Resources Table

**Table:** 

REAGENT or RESOURCE	SOURCE	IDENTIFIER
**Antibodies**		

CD41 clone eBioMWReg30	eBioscience	Cat#13-0411-82
		RRID:AB_763484
CD45 clone 30-F11	eBioscience	Cat#13-0451-85
		RRID:AB_466447
streptavidin-conjugated PerCP	Biolegend	Cat#405213
E-cad-eFluor660	eBioscience	Cat#50-3249-82
		RRID:AB_11040003

**Chemicals, Peptides, and Recombinant Proteins**		

DNase I	Roche	Cat#4716728001
Isoflurane	Pharmachemie BV, Haarlem, Netherlands	Cat# 45.112.110
Histopaque-1077	Sigma	Cat#10771
DMEM/F12 + GlutaMAX	Invitrogen Life Technologies	Cat#10565018
TH Liberase	Roche	Cat#5401151001
Trizol	Invitrogen Life Technologies	Cat#15596018
Chloroform	Sigma	Cat#77617-500ml
Sucrose	Sigma	Cat#S0389-500G
L-Lysine	Sigma	Cat#W384720-100G-K
Sodium Dihydrogen Phosphate Dihydrate	Fischer Scientific	Cat#RS228270015
Paraformaldehyde	Alfa Aesar	Cat#43368
Sodium metaperiodate	Merck	Cat#106597
Trypsin	Sigma	Cat# T1426
Collagenase A	Roche	Cat# 10103578001
BME	PathClear	Cat# 3533-005-02
B27	Thermo Fisher Scientific	Cat# 17504044

**Critical Commercial Assays**		

High-Capacity cDNA Reverse Transcription Kit	Applied Biosystems	Cat#4368814
Power SYBR Green PCR Master Mix	Applied Biosystems	Cat#A25742

**Experimental Models: Cell Lines**		

1805473 organoid line *MMTV*-PyMT; *fsp1*-Cre; *R26*-mTmG; E-cad-mCFP	This manuscript	N/A
1814468 organoid line *MMTV*-PyMT; *fsp1*-Cre; *R26*-mTmG; E-cad-mCFP	This manuscript	N/A
1926833 organoid line MMTV-PyMT; *fsp1*-Cre; *R26*-mTmG; E-cad-mCFP	This manuscript	N/A

**Experimental Models: Organisms/Strains**		

NOD-scid Il2ry^null^B2m^null^	Jackson Laboratory	Stock No:010636
*MMTV*-PyMT	Jackson Laboratory	Stock No:002374
*fsp1*-Cre	Jackson Laboratory	Stock No:012641
*R26*-mTmG in house backcrossed to FVB/N	Jackson Laboratory	Stock No:007676
E-cad-mCFP	Gift from Hans Clevers	N/A

**Oligonucleotides**		

qPCR primers	Beerling et al., 2016	see Table S2

**Software and Algorithms**		

Prism v7	Graphpad	https://www.graphpad.com/scientific-software/prism/
Excel 2010	Microsoft Office	https://products.office.com/en/excel
FlowJo v10	TreeStar	https://www.flowjo.com/solutions/flowjo/downloads
LasX	Leica Microsystems	https://www.leica-microsystems.com/products/microscope-software/p/leica-las-x-ls/

### Lead Contact and Materials Availability

All mouse organoid generated in this study are available from the Lead Contact with a completed Materials Transfer Agreement. Further information and requests for resources and reagents should be directed to and will be fulfilled by the Lead Contact, Jacco van Rheenen (j.v.rheenen@nki.nl).

### Experimental Model and Subject Details

*R26*-mTmG C57BL/6J were purchased from Jackson Laboratory and were backcrossed in house to FVB. Further, *MMTV*-PyMT (FVB) mice and *fsp1*-Cre (Balb-c) were purchased from Jackson Laboratory and E-cad-mCFP mice were a gift from Hans Clevers. Mice were crossed *MMTV*-PyMT; *fsp1*-Cre; *R26*-mTmG; E-cad-mCFP transgenic mice.

As acceptors for orthotopic transplantation and tail vein injection 8 to 16 weeks old female NOD-scid Il2ry^null^B2m^null^ mice (referred in the text to as NSG-β2 m^−/−^ mice) were used. All animal experiments were approved by the Animal Welfare Committee of the NKI, in accordance with national guidelines. All animals were maintained in the animal department of the NKI, housed in individually ventilated cage (IVC) systems under specific pathogen-free conditions and received food and water *ad libitum*.

### Method Details

#### Isolation and culturing of donor mouse organoids

*MMTV-PyMT; fsp1*-Cre; R26-mTmG; E-cad-mCFP transgenic mice spontaneously developed mammary tumors at the age of 8-14 wks. Upon tumor formation, mice were sacrificed and mammary tumor organoids were isolated from three independent donors. Mammary tumors were minced and enzymatically digested gently shaken for 30 min at 37 C in digestion mix (0.2% trypsin (from bovine pancreas, Sigma) and 0.2% collagenase A (Roche)). The digested tumors were spun down and cell fragments were ebedded in BME (RGF BME type 2 pathClear). Mammary tumor organoid medium contained DMEM/F12 Glutamax (GIBCO), 2% B27 (Invitrogen), 10 ng/ml FGF. In order to ensure to start from a pure epithelial population, RFP^+^, E-cad-mCFP^HI^ cells were selected by FACS sorting and expanded by culturing as tumor organoid lines.

#### FACS sorting of primary mouse material

Deoxygenated blood was withdrawn from the right cardiac ventricle while the mice were under anesthesia (1.5% isoflurane). Red blood cells were depleted by NH4Cl treatment. The remaining circulating tumor cells and immune cells were collected (spun down, 4 minutes 500 RCF at RT).

Orthotopic mammary tumors were collected and minced on ice using sterile scalpels, followed by digestion in PBS supplemented with 25 μg/ml DNase I (Roche) and 5 Wünsch units TH Liberase /ml (Roche) at 37 C for 35 min. Digested cell clumps were filtered through a 70 μm filter (BD Falcon) while adding DMEM/F12 + GlutaMAX and spun down for 4 min at 500 RCF at 4 C. Pellets were resuspended in 5mM EDTA/PBS, and live cells were selected using a Ficoll gradient (Histopaque-1077, Sigma) (30 min at 400 RCF at RT, break 0). Cells were washed once in 5 mM EDTA/PBS and centrifuged (4 min at 500 RCF at RT) before proceeding with antibody labeling.

Tumor cells and blood cells were blocked in FACS buffer supplied with 20% normal goat serum (GIBCO) for 10 min on ice before labeling with the following antibodies: E-cad-eFluor660 (DECMA-1, eBioscience), biotin-conjugated anti-mouse CD41 clone eBioMWReg30 (eBioscience, cat. no. 13-0411-82) and anti-mouse CD45 clone 30-F11 (eBioscience, cat. no. 13-0451-85). Secondary labeling was performed using streptavidin-conjugated PerCP (Biolegend). Cells were sorted on a FACS Aria II Special Ordered Research Product (BD Biosciences). A broad FSC/SSC gate was followed by gates excluding doublets. Afterward, immune cells and megakaryocytes were excluded, based on staining for CD41 and CD45 in a dump channel. Tumor cells were subdivided as either RFP^+^ or GFP^+^ and further stringently gated for either absence of endogenous E-cad-mCFP expression and antibody staining (E-cad^LO^) or presence of high expression of E-cadherin and staining for Ab (E-cad^HI^ tumor cells). Data were manually analyzed with FlowJo.

#### RNA isolation

RNA was isolated using Trizol reagent (Invitrogen Life Technologies) according to the manufacturer’s protocol. The amount and purity of isolated RNA was analyzed by the Nanodrop spectrophotometer (Wilmington, DE, USA).

#### cDNA preparation and qPCR

cDNA was prepared using High-Capacity cDNA Reverse Transcription Kit (Applied Biosystems) according to the manufacturer’s protocol. Sequences of used primers can be found below. qPCR was performed using Power SYBR Green PCR Master Mix (Applied Biosystems). Thermal cycle conditions used for all qPCR reactions were as follows: 5 min at 95°C, followed by 40 cycles consisting of denaturation for 30 s at 95°C, annealing for 30 s at 60°C, and extension for 1 min at 72°C. PCR reactions were concluded with incubation for 10 min at 72°C to complete the extension of all synthesized products.

#### Tail vein injection

FACS-sorted cells were resuspended in sterile PBS and injected in the tail vein of acceptor mice (100 μl per mouse). 3 months after injection acceptor mice were sacrificed and lungs were inspected for presence of metastases under a fluorescence-stereo microscope (Leica). Tissues were fixed using periodate-lysine-4% paraformaldehyde (PLP) buffer overnight at 4°C, incubated in 30% sucrose overnight at 4°C and embedded in Tissue Freezing medium (Leica Biosystems). Organs were cryo-sectioned (50 μm) and metastases were imaged with an inverted Leica TCS SP8 confocal microscope. All images were collected in 12 bit with 25X water immersion objective (HC FLUOTAR L N.A. 0.95 W VISIR 0.17 FWD 2.4 mm).

### Quantification and Statistical Analysis

Data were analyzed using Prism v7 (GraphPad). Statistical significance for relation was assessed by linear regression. Data were normalized in some cases. And either single values per mouse or mean ± standard division are plotted throughout the manuscript.

### Data and Code Availability

This study did not generate any unique datasets or code.

## Supplemental Information

Document S1. Figure S1 and Tables S1 and S2

Document S2. Article plus Supplemental Information

## References

[bib1] Aiello N.M., Brabletz T., Kang Y., Nieto M.A., Weinberg R.A., Stanger B.Z. (2017). Upholding a role for EMT in pancreatic cancer metastasis. Nature.

[bib2] Aiello N.M., Maddipati R., Norgard R.J., Balli D., Li J., Yuan S., Yamazoe T., Black T., Sahmoud A., Furth E.E. (2018). EMT subtype influences epithelial plasticity and mode of cell migration. Dev. Cell.

[bib3] Beerling E., Seinstra D., de Wit E., Kester L., van der Velden D., Maynard C., Schäfer R., van Diest P., Voest E., van Oudenaarden A. (2016). Plasticity between epithelial and mesenchymal states unlinks EMT from metastasis-enhancing stem cell capacity. Cell Rep..

[bib4] Bill R., Christofori G. (2015). The relevance of EMT in breast cancer metastasis: correlation or causality?. FEBS Lett..

[bib5] Brabletz T., Kalluri R., Nieto M.A., Weinberg R.A. (2018). EMT in cancer. Nat. Rev. Cancer.

[bib6] Cano A., Pérez-Moreno M.A., Rodrigo I., Locascio A., Blanco M.J., del Barrio M.G., Portillo F., Nieto M.A. (2000). The transcription factor snail controls epithelial-mesenchymal transitions by repressing E-cadherin expression. Nat. Cell Biol..

[bib7] Diepenbruck M., Christofori G. (2016). Epithelial-mesenchymal transition (EMT) and metastasis: yes, no, maybe?. Curr. Opin. Cell Biol..

[bib8] Fischer K.R., Durrans A., Lee S., Sheng J., Li F., Wong S.T., Choi H., El Rayes T., Ryu S., Troeger J. (2015). Epithelial-to-mesenchymal transition is not required for lung metastasis but contributes to chemoresistance. Nature.

[bib9] Fischer K.R., Altorki N.K., Mittal V., Gao D. (2017). Fischer et al. reply. Nature.

[bib10] Hanahan D., Weinberg R.A. (2011). Hallmarks of cancer: the next generation. Cell.

[bib11] Jolly M.K., Ware K.E., Gilja S., Somarelli J.A., Levine H. (2017). EMT and MET: necessary or permissive for metastasis?. Mol. Oncol..

[bib12] Lin E.Y., Jones J.G., Li P., Zhu L., Whitney K.D., Muller W.J., Pollard J.W. (2003). Progression to malignancy in the polyoma middle T oncoprotein mouse breast cancer model provides a reliable model for human diseases. Am. J. Pathol..

[bib13] Mani S.A., Guo W., Liao M.J., Eaton E.N., Ayyanan A., Zhou A.Y., Brooks M., Reinhard F., Zhang C.C., Shipitsin M. (2008). The epithelial-mesenchymal transition generates cells with properties of stem cells. Cell.

[bib14] Nieto M.A., Huang R.Y., Jackson R.A., Thiery J.P. (2016). EMT: 2016. Cell.

[bib15] Pastushenko I., Brisebarre A., Sifrim A., Fioramonti M., Revenco T., Boumahdi S., Van Keymeulen A., Brown D., Moers V., Lemaire S. (2018). Identification of the tumour transition states occurring during EMT. Nature.

[bib16] Reichert M., Bakir B., Moreira L., Pitarresi J.R., Feldmann K., Simon L., Suzuki K., Maddipati R., Rhim A.D., Schlitter A.M. (2018). Regulation of epithelial plasticity determines metastatic organotropism in pancreatic cancer. Dev. Cell.

[bib17] Thiery J.P., Acloque H., Huang R.Y., Nieto M.A. (2009). Epithelial-mesenchymal transitions in development and disease. Cell.

[bib18] Tsai J.H., Donaher J.L., Murphy D.A., Chau S., Yang J. (2012). Spatiotemporal regulation of epithelial-mesenchymal transition is essential for squamous cell carcinoma metastasis. Cancer Cell.

[bib19] Yang J., Mani S.A., Donaher J.L., Ramaswamy S., Itzykson R.A., Come C., Savagner P., Gitelman I., Richardson A., Weinberg R.A. (2004). Twist, a master regulator of morphogenesis, plays an essential role in tumor metastasis. Cell.

[bib20] Ye X., Brabletz T., Kang Y., Longmore G.D., Nieto M.A., Stanger B.Z., Yang J., Weinberg R.A. (2017). Upholding a role for EMT in breast cancer metastasis. Nature.

[bib21] Yeung K.T., Yang J. (2017). Epithelial-mesenchymal transition in tumor metastasis. Mol. Oncol..

[bib22] Yu M., Bardia A., Wittner B.S., Stott S.L., Smas M.E., Ting D.T., Isakoff S.J., Ciciliano J.C., Wells M.N., Shah A.M. (2013). Circulating breast tumor cells exhibit dynamic changes in epithelial and mesenchymal composition. Science.

[bib23] Zheng X., Carstens J.L., Kim J., Scheible M., Kaye J., Sugimoto H., Wu C.C., LeBleu V.S., Kalluri R. (2015). Epithelial-to-mesenchymal transition is dispensable for metastasis but induces chemoresistance in pancreatic cancer. Nature.

