# Supplementary material for: Fsp1-Mediated Lineage Tracing Fails to Detect the Majority of Disseminating Cells Undergoing EMT
Source: Cell Rep. 2019 Nov 26;29(9):2565–2569.e3. doi: 10.1016/j.celrep.2019.10.107 (PMC6899519; doi:10.1016/j.celrep.2019.10.107)
Supplement: Document S1. Figure S1 and Tables S1 and S2 [file mmc1.pdf]

Cell Reports, Volume *29*

## Supplemental Information

# *Fsp1*-Mediated Lineage Tracing Fails to Detect the Majority of Disseminating Cells Undergoing EMT

**Laura Bornes, Roan Hugo van Scheppingen, Evelyne Beerling, Tim Schelfhorst, Saskia Inge Johanna Ellenbroek, Danielle Seinstra, and Jacco van Rheenen**

**A**

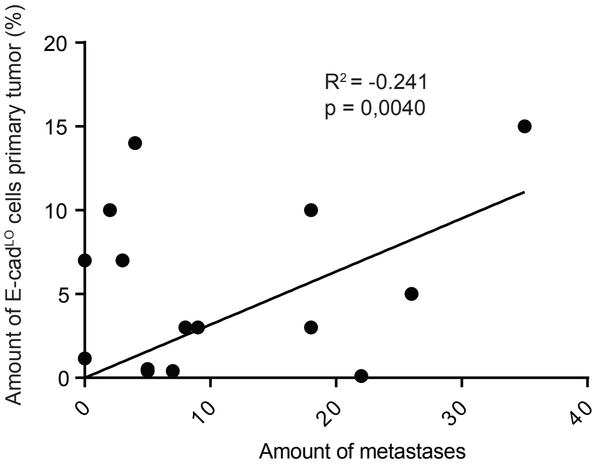

Amount of E-cad^LO cells primary tumor (%)
20
15
10
5
0
R² = -0.241
p = 0,0040
0
10
20
30
40
Amount of metastases

**Figure S1:** Mesenchymal E-cad$^{LO}$ population in the primary tumor is proportional to amount of metastases. Related to Figures 1 and 2.

(A) The percentage of E-cad$^{LO}$ cells in the primary tumor relates to the amount of metastases. (p=0,0040)

| Mouse | Donor tumor | time of tumor growth wks | Macro Metastases >99.9 RFP$^+$ | >99.9 GFP$^+$ | total | FACS analysis | Primary tumor RFP$^+$ | RFP$^+$GFP$^+$ | GFP$^+$ | Ecad$^{HI}$ | Ecad$^{LO}$ | CTCs blood (ul) | total amount of CTCs | RFP$^+$ | RFP$^+$GFP$^+$ | GFP$^+$ | Ecad$^{HI}$ | Ecad$^{LO}$ |
|---|---|---|---|---|---|---|---|---|---|---|---|---|---|---|---|---|---|---|
| 1 | 1 | 8,4 | 26 | 0 | 26 | YES | 99,68 | 0,19 | 0,13 | 94 | 5 | 950 | 501 | 99,6 | 0,2 | 0,2 | 92,8 | 7,2 |
| 2 | 1 | 7,4 | 8 | 1 | 9 | YES | 99,92 | 0,06 | 0,02 | 96 | 3 | 500 | 0 | 0 | 0 | 0 | 0 | 0 |
| 3 | 1 | 9,4 | 9 | 0 | 9 | YES | 99,46 | 0,25 | 0,29 | 96 | 3 | 850 | 9 | 100 | 0 | 0 | 55,6 | 44,4 |
| 4 | 1 | 8,1 | 18 | 0 | 18 | YES | 99,56 | 0,32 | 0,12 | 89 | 10 | 350 | 3 | 100 | 0 | 0 | 66,7 | 33,3 |
| 5 | 1 | 6,4 | 0 | 0 | 0 | YES | 99,86 | 0,08 | 0,06 | 92 | 7 | 850 | 2 | 100 | 0 | 0 | 50 | 50 |
| 6 | 1 | 6,0 | 3 | 0 | 3 | NO | | | | | | | | | | | | |
| 7 | 1 | 4,3 | 0 | 0 | 0 | NO | | | | | | | | | | | | |
| 8 | 1 | 6,0 | 35 | 0 | 35 | YES | 99,79 | 0,05 | 0,16 | 84 | 15 | 650 | 0 | 0 | 0 | 0 | 0 | 0 |
| 9 | 1 | 4,0 | 5 | 0 | 5 | NO | | | | | | | | | | | | |
| 10 | 1 | 6,3 | 0 | 0 | 0 | NO | | | | | | | | | | | | |
| 11 | 2 | 7,1 | 3 | 0 | 3 | YES | 95,00 | 2,27 | 2,67 | 92 | 7 | 1100 | 0 | 0 | 0 | 0 | 0 | 0 |
| 12 | 2 | 7,3 | 2 | 0 | 2 | NO | | | | | | | | | | | | |
| 13 | 2 | 8,1 | 0 | 0 | 0 | NO | | | | | | | | | | | | |
| 14 | 2 | 9,1 | 2 | 0 | 2 | YES | 98,96 | 0,42 | 0,61 | 89 | 10 | 1050 | 0 | 0 | 0 | 0 | 0 | 0 |
| 15 | 2 | 7,1 | 8 | 0 | 8 | NO | | | | | | | | | | | | |
| 16 | 2 | 6,1 | 8 | 0 | 8 | YES | 98,68 | 0,20 | 1,13 | 97 | 3 | 900 | 0 | 0 | 0 | 0 | 0 | 0 |
| 17 | 3 | 7,1 | 18 | 0 | 18 | YES | 99,5 | 0,45 | 0,05 | 97 | 3 | 700 | 0 | 0 | 0 | 0 | 0 | 0 |
| 18 | 3 | 8,0 | 5 | 0 | 5 | YES | 99,90 | 0,08 | 0,02 | 99,6 | 0,4 | 850 | 1 | 100 | 0 | 0 | 100 | 0 |
| 19 | 3 | 7,9 | 7 | 0 | 7 | YES | 99,8 | 0,16 | 0,04 | 99,6 | 0,4 | 350 | 8 | 100 | 0 | 0 | 87,5 | 12,5 |
| 20 | 3 | 7,9 | 0 | 0 | 0 | YES | 99,90 | 0,09 | 0,01 | 98,85 | 1,15 | 750 | 17 | 100 | 0 | 0 | 70,6 | 29,4 |
| 21 | 3 | 7,1 | 22 | 0 | 22 | YES | 99,7 | 0,29 | 0,01 | 99,9 | 0,1 | 550 | 3 | 100 | 0 | 0 | 66,7 | 33,3 |
| 22 | 3 | 7,7 | 4 | 0 | 4 | YES | 99,50 | 0,45 | 0,05 | 86 | 14 | 650 | 46 | 100 | 0 | 0 | 78,3 | 21,7 |
| 23 | 3 | 7,0 | 5 | 0 | 5 | YES | 99,7 | 0,17 | 0,13 | 99,6 | 0,52 | 700 | 0 | 0 | 0 | 0 | 0 | 0 |
| Average | | | 8,2 | 0,0 | 8,2 | | 99,3 | 0,3 | 0,3 | 94,3 | 5,2 | | 36,9 | | 0,0 | 0,0 | 74,2 | 25,8 |

**Tabel S1:** Experimental summary of recipient mice. Related to Figure 1 and 2.

| Oligonucleotides | | | |
|---|---|---|---|
| **name** | **sequence** | **Source** | **Identifier** |
| GAPDH fwr | 5'-GGT CCT CAG TGT AGC CCA AG-3' | Beerling *et al.,* 2016 | N/A |
| GAPDH rev | 5'-AAT GTG TCCGTC GTG GAT CT-3' | Beerling *et al.,* 2016 | N/A |
| E-cad fwr | 5'-GCT TCA GTT CCG AGG TCT AC-3' | Beerling *et al.,* 2016 | N/A |
| E-cad rev | 5'-GCC AGT GCA TCC TTC AAA TC-3' | Beerling *et al.,* 2016 | N/A |
| Vim fwr | 5'-GAG GAG ATG CTC CAG AGA GA-3' | Beerling *et al.,* 2016 | N/A |
| Vim rev | 5'-TCC TGC AAG GAT TCC ACT TT-3' | Beerling *et al.,* 2016 | N/A |
| FN fwr | 5'-GGT GTC CGA TAC CAG TGT TAC-3' | Beerling *et al.,* 2016 | N/A |
| FN rev | 5'-TCT CCG TGA TAA TTA CTT GGA CAG-3' | Beerling *et al.,* 2016 | N/A |
| Zeb1 fwr | 5'-TGG CAA GAC AAC GTG AAA GA-3' | Beerling *et al.,* 2016 | N/A |
| Zeb1 rev | 5'-AAC TGG GAA AAT GCA TCT GG-3' | Beerling *et al.,* 2016 | N/A |
| N-cad fwr | 5'-GTG GAG GCT TCT GGT GAA AT-3' | Beerling *et al.,* 2016 | N/A |
| N-cad rev | 5'-GGC TCG CTG CTT TCA TAC T-3' | Beerling *et al.,* 2016 | N/A |
| Slug fwr | 5'-TGA TGC CCA GTC TAG GAA AT-3' | Beerling *et al.,* 2016 | N/A |
| Slug rev | 5'-AGT GAG GGC AAG AGA AAG G-3' | Beerling *et al.,* 2016 | N/A |

**Tabel S2:** Oligonucleotides used in this study. Related to Figure 1 D.
